# Smart Actuators Based on External Stimulus Response

**DOI:** 10.3389/fchem.2021.650358

**Published:** 2021-05-31

**Authors:** Qinchao Zheng, Chenxue Xu, Zhenlin Jiang, Min Zhu, Chen Chen, Fanfan Fu

**Affiliations:** ^1^ College of Chemistry and Chemical Engineering, Research Center for Advanced Mirco- and Nano-Fabrication Materials, Shanghai University of Engineering Science, Shanghai, China; ^2^ Science and Technology on Advanced Ceramic Fibers and Composites Laboratory, National University of Defense Technology, Changsha, China; ^3^ School of Materials Science and Engineering, Nanyang Technological University, Singapore, Singapore

**Keywords:** smart actuators, single stimuli, multi stimuli, drive response, functional nanomaterials

## Abstract

Smart actuators refer to integrated devices that are composed of smart and artificial materials, and can provide actuation and dampening capabilities in response to single/multi external stimuli (such as light, heat, magnetism, electricity, humidity, and chemical reactions). Due to their capability of dynamically sensing and interaction with complex surroundings, smart actuators have attracted increasing attention in different application fields, such as artificial muscles, smart textiles, smart sensors, and soft robots. Among these intelligent material, functional hydrogels with fiber structure are of great value in the manufacture of smart actuators. In this review, we summarized the recent advances in stimuli-responsive actuators based on functional materials. We emphasized the important role of functional nano-material-based additives in the preparation of the stimulus response materials, then analyzed the driving response medium, the preparation method, and the performance of different stimuli responses in detail. In addition, some challenges and future prospects of smart actuators are reported.

## Introduction

With revolutionary developments of nanomaterials and bionics, smart actuators in response to natural muscles has attracted considerable attention in the last decade. Taking advantage of those functional materials with controllable shape or volume changes under external stimuli (such as light, heat, electricity, magnetism, humidity, and chemistry), smart actuators can convert such stimuli into mechanical energy in response to environmental stimuli (Xu et al., 2015; Song et al., 2016; Shin et al., 2018; Li J. et al., 2019; Chortos et al., 2019; Sturm et al., 2019). Smart actuators have a wide range of application prospects in the fields of biomedicine, bionic robots, and smart medicine micro/nanomanipulators (Zang et al., 2013; Yao et al., 2015; Santhiago et al., 2016; Hua et al., 2018; Power et al., 2018; Jia H. et al., 2019). They are derived from smart materials with sensing and executive functions, as first proposed by Toshiyoshi and Newham in the late 1980s. Subsequently, Finkelmann (Finkelmann et al., 2001) and Li (Li et al., 2003) used azobenzene-containing polymer liquid crystal hydrogels to prepare smart responsive materials that can bend under light stimulation. Yu et al. prepared liquid crystal polymer smart materials that can achieve controlled directional photochemical bending memory changes under ultraviolet (UV) irradiation (Yu et al., 2003).

**FIGURE 1:**
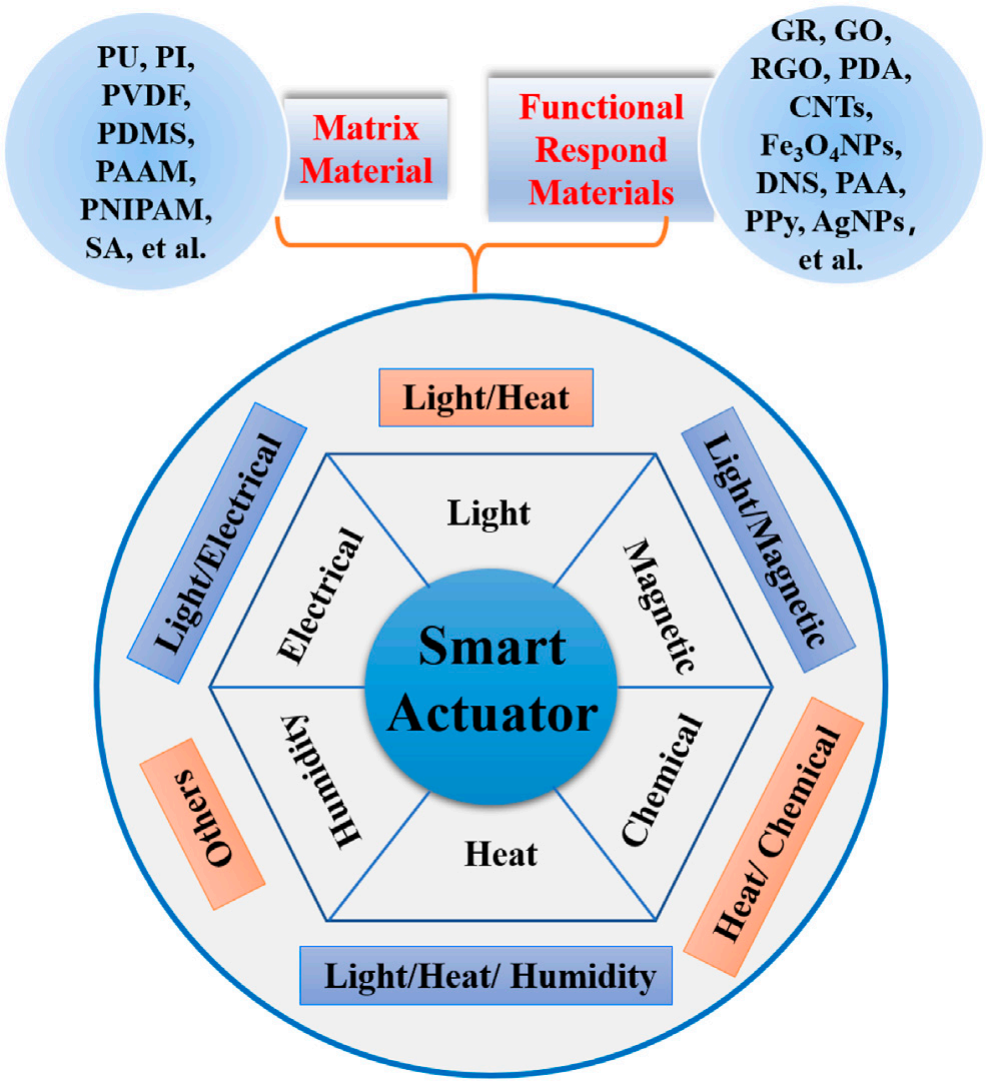
Classification and composition of smart actuators based on single/multiple responses to different stimuli.

Since the structure of nanomaterials consists of crystalline units and interfacial units, their quantum size effect and surface effect make them far superior to ordinary materials in terms of physicochemical properties (Hasan, 2020), including melting point, magnetic properties, optical properties, capacitive properties, and water solubility. So, smart actuators have evolved from initial light stimulus response to an exciter driven by single or multiple responses under different stimuli due to combine with functional nanomaterials. However, the classification boundaries of the actuator are still unclear. From the perspective of morphological structure, they can be classified into fiber (1D), membrane (2D), and block (3D) classes. In terms of stimulus responsiveness, they can be divided into single stimulus and multiple stimuli. This review discusses recent advances in smart actuators with different single stimulus and multiple stimuli response. We mainly focus on the recent progress of single and multiple responses smart actuators in points of material designs, fabrication methods, and performance (Figure 1). Finally, we discuss the current applications and possible new fields of interest for these smart actuators.

## Single Stimulus Response Smart Actuators

For smart actuators, the most critical attributes (Huang et al., 2012) that should be simultaneously performed are perception, processing, and response capabilities. Internal molecules perform the corresponding motion processing by sensing the external stimuli and causing the material drive and response, such as heat shrinkage and cold bending (Leng et al., 2011). For single stimulus response smart actuators, a better targeted response and timely shape change can be accomplished if there is only a single variation in the environment. In addition, actuators responsive to a single stimulus have a high response accuracy and stable response to remote control in actual applications that have more advanced preparation technologies.

### Smart Actuators Based on Light Stimulation Response

Light stimulation is one of the most basic and direct methods for smart actuators, especially in single stimulus response research, owing to its several advantages, such as fast stimulus response, high rate of change in drive performance, and good stability. Photochromic molecules play a major role in light-responsive actuators, capturing light signals and translating these to useful property changes, thereby achieving changes in geometric size or shape and structure, and showing macroscopic motion characteristics (Jiang et al., 2006). This is similar to light-driven mechanisms in nature.

**FIGURE 2:**
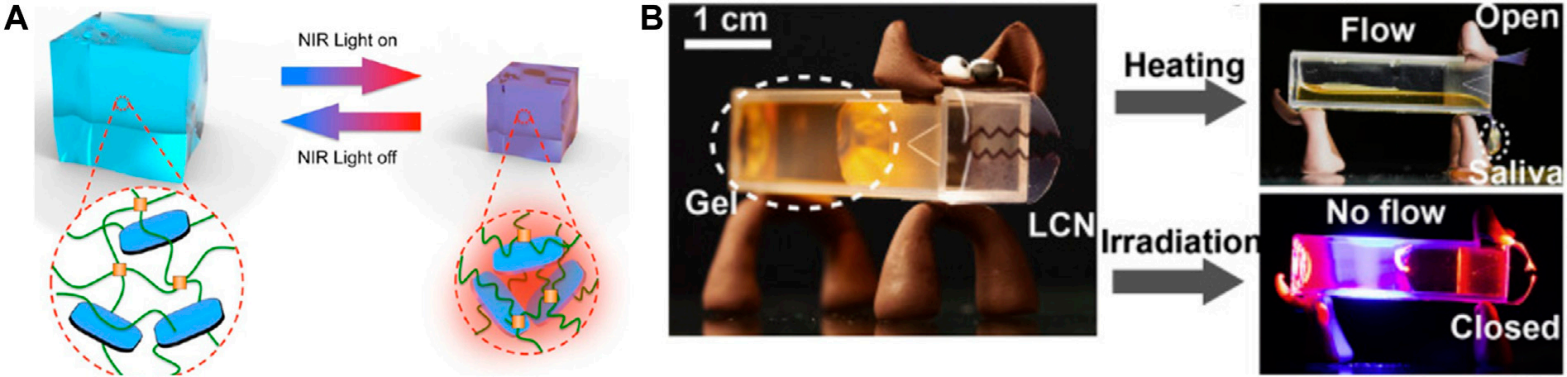
**(A)** Reversible contraction of PNIPAM/GO nanocomposite hydrogels actuator responds to IR light stimulation. Reproduced from Shi et al. (2015) with permission of American Chemical Society. **(B)** Side view of the original dog after incubation at 50°C for 27 min, showing salivation (gel dripping). Reproduced from Zeng et al. (2020) with permission of Elsevier.

Based on the characteristics of a fiber structure mimicking human muscles, CNTs were mixed with PU solution to form electrospinning precursor, and the so-made yarns can be triggered by NIR (Meng et al., 2019). Due to the high heat absorption property of CNTs, CNTs can enable the yarns to efficiently absorb NIR and radiate heat, which induces the fast temperature change that leads to the contraction/expansion motions along the axial direction. So, the yarns relaxed immediately, showing fast thermal radiation speed and the maximum contractive actuation of 6.7% after 6 s exposed to the NIR light, and returned to its initial state at 16 s. Although the radiation speed is fast, the deformation efficiency is smaller, and it is also a common problem of fiber-based smart actuators. Different from fiber-based smart actuators, the common poor ductility of hydrogel smart actuators has been hindering the further application of light responsive actuators (Yu and Ikeda, 2010), NIR light responsive PNIPAM/GO composite hydrogels with ultra-high tension were prepared by combining different polymerization methods with UV polymerization (Shi et al., 2015) and 3D printing technology (Zhang et al., 2019). Combination of the GO and thermoresponsive PNIPAM polymeric networks provides the hydrogels with an excellent NIR light-responsive property, and the physical cross-linking of the GO increases the toughness of the nanocomposite hydrogel networks. Turning on or off the NIR light respectively caused the contraction and swelling of the actuator, which shows in Figure 2A. Furthermore, the fast and reversible NIR response characteristics of the actuator were realized by changing the GO content and irradiation time of NIR light. In contrast, Kim et al. prepared light-responsive bilayer hydrogel actuators by crosslinking PNIPAM/ RGO composite hydrogels as the active layer and poly(acrylamide) hydrogels as the passivation layer. The volume of the active layer decreased through light simulation, while the passivated layer maintained its original size, and the asymmetric volume size induced the full bending motion of the bilayer actuator (Kim et al., 2016). Similarly, a bilayer composed of RGO and elastin-like polypeptides can be driven by an NIR laser, achieving 60° bending in 1 s, and recovering 84% in 10s (Wang et al., 2013). Based on the application characteristics of liquid crystal networks in remote and wireless control of the bending of actuators, the photopolymerization of monoacrylate, diacrylate mesogens, and azobenzene chromophores were used to form light responsive switch molecules, and a non-binding multifunctional light-driven soft robot was also prepared from the different relaxed states of the curled shapes and light sensitivity (Pilz da Cunha et al., 2020). Heat was released through the isomerization process under light stimulation, thereby driving displacement of 20 mm, and exhibiting a transportation behavior. In a recent example reported that autonomous walking and “salivation” behavior can also be achieved in artificial dogs under periodic light stimulation (Zeng et al., 2020), as shown in Figure 2B. In short, light-responsive smart actuators are compelling because they can be remotely and accurately controlled, rapidly modulated, and easily focused on microscale drive field.

### Smart Actuators Based on Electrical Stimulation Response

There are many types of materials with flexible or soft materials that can convert electrical energy into mechanical energy, including some polymers, gels, and even CNTs. Smart actuators driven by electrical signals can easily adjust its motion amplitude. Among the electrical stimulation responsive actuators, electroactive polymers are one of the most widely studied materials, which can change size or deform under electrical stimulation. Furthermore, this electroactive polymer can not only exhibit considerable strain and stress, strong mechanical flexibility, but also can provide the largest drive change in volume (Zhao et al., 2016). So, some of the biggest research breakthroughs are reported in artificial muscles (Takemura et al., 2008) and soft robots (Must et al., 2015; Nhat and Truong Thinh, 2015).

**FIGURE 3:**
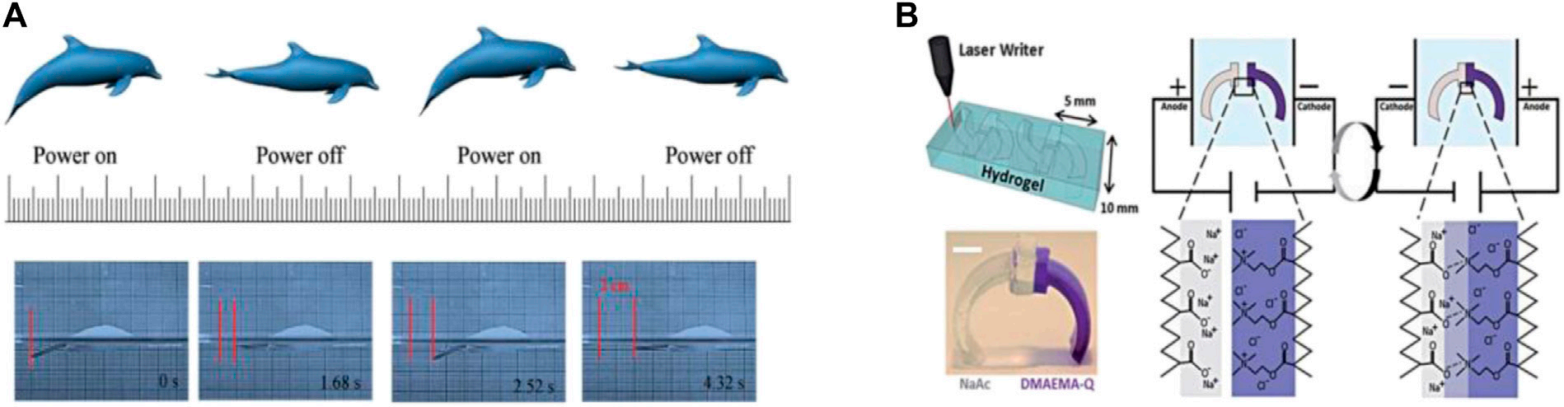
**(A)** fish-like robot swimming, when the power is on or off, the “tail” bends down or up, then the fish-like robot will swim. Reproduced from Xiao et al. (2016) with permission of WILEY-VCH. **(B)** Based on the gel electric stimulation of the smart actuator and walking under the electric field drive. Reproduced from Morales et al. (2014) with permission of Royal Society of Chemistry.

Xiao et al. reported a electromechanical bimorph actuator constituted by a GR layer and a PVDF layer (Xiao et al., 2016), and taking advantage of the differences in coefficient of thermal expansion between the two layers and the converse piezoelectric effect and electro strictive property of the PVDF layer, the fish-like robots could swim at a speed of 5.02 mm/s applied the voltage of 0-13 V and the frequency of 0.4 Hz, as shown in Figure 3A. Morales et al. combined two oppositely deforming polyelectrolyte hydrogels to create a walker (Morales et al., 2014). Under an electric field of 5 V/cm constantly changing between positive and negative electrodes, the hydrogel chain moved across the cation/anion gel interface to the oppositely charged electrode. With this, the adhesion of the polyion complex became stronger, thereby promoting the separation by reversing the electric field and resulting in a walking motion (Figure 3B). Electrical stimulus actuators generally have low energy conversion efficiency owing to the lack of active units in their microstructure. Lu et al. achieved a 6.03% energy conversion rate and strain capacity of 16.45%, which are significantly higher than that of other CNTs with a graphene actuator voltage of 2.5 V (Lu C. et al., 2018). Electrical stimulus responsive actuators have a wide frequency spindle that allows it to bend at 0.1–30 Hz. Recently, the coiled GO/CNTs yarns made by the biscrolling method can produce 19% maximum tensile actuation (Hyeon et al., 2019), and compared with an original CNT artificial muscle with a work capacity of 2.6 J/g, GO/CNTs actuator can produce approximately twice the tensile actuation force at the same voltage. Therefore, electric stimulus smart actuators can be used as an artificial muscle to imitate the shape deformation of muscle cells. In order to solve the limited multi-function integration problem of most actuators, the large amount of PANI nanoparticles on the surface of GP paper-like actuator was reported, which can provide large pseudocapacitance as power supply units in soft robots (Weng et al., 2020). It had the areal specific capacitance of 402.5 mF/cm^2^ and bending curvature of 1.03 cm^-1^ when GP was used for the component layer of actuator and supercapaction electrodes. Furthermore, several researchers are also working on biocompatible and multi-functional silk fibroin-based hydrogels (Xu et al., 2019; He et al., 2020). Based on the current effect of electrical stimulation, smart actuators controlled by a variable current (Winchester, 2009) are also essential in capturing nanoparticles. So, the electrical stimulation actuators are compatible with electronic devices and batteries, and if a lower voltage drive can be achieved in the future, it is easy to integrate them with power supplies for using in sensors and industrial automation field.

### Smart Actuators Based on Humidity Stimulation Response

**FIGURE 4:**
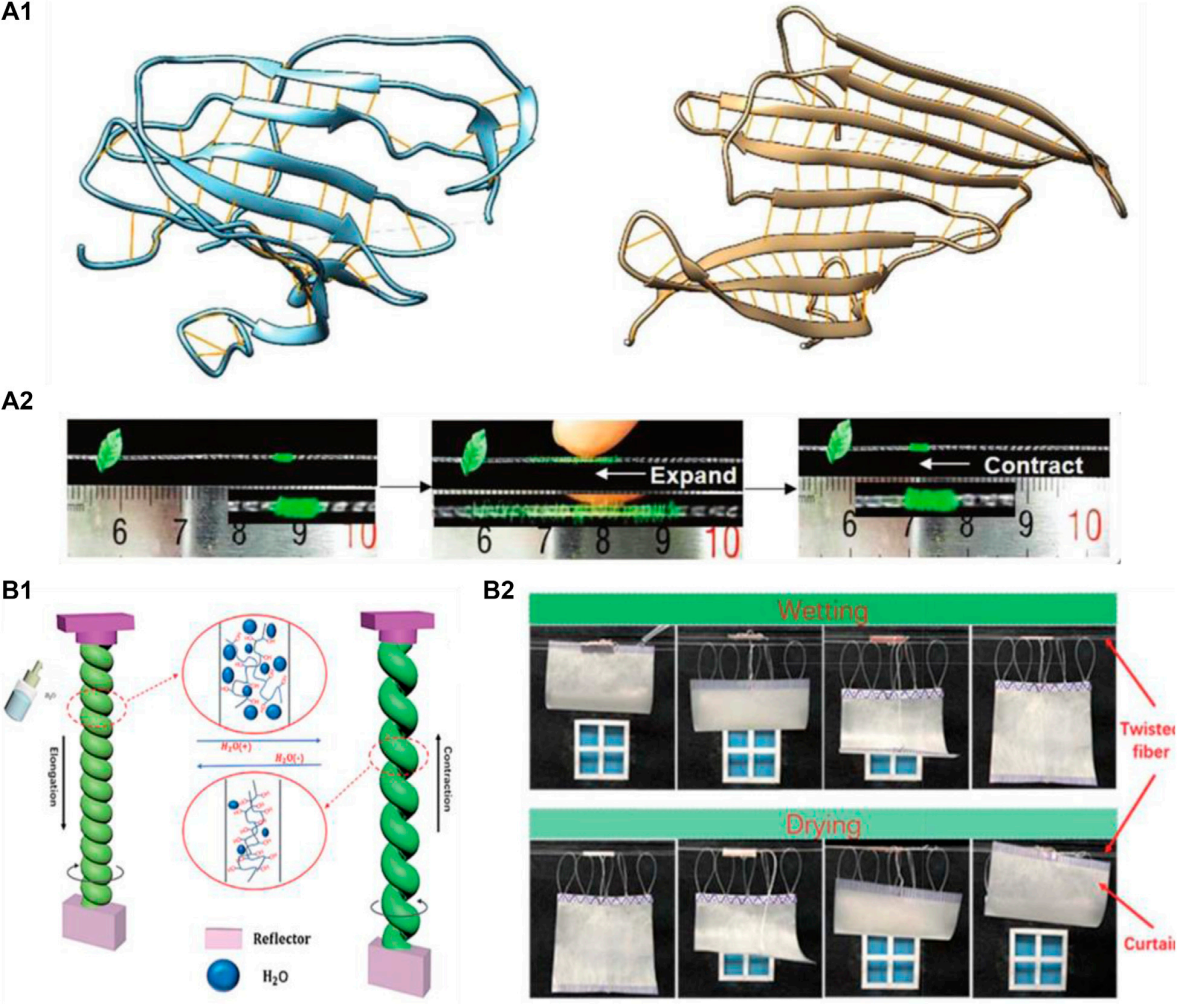
**(A1)** Molecular dynamic simulation of the proteins before and after water molecules added, the loop structure undergoes transition into a protein secondary structure, and the structure becomes more regular. **(A2)** a robotic “caterpillar” walking on a barbed wire by moisture stimulation. Reproduced from Jia T. et al. (2019) with permission of WILEY-VCH. **(B1)** the twisted fiber rapid swelling occurred under water stimulation, leading to fast rotation. After the water evaporated, the fiber quickly returned to its original state. **(B2)** Schematic diagram of the smart rainy curtain. Reproduced from Wang et al. (2018c) with permission of Royal Society of Chemistry.

As we all know, humidity stimulus response smart actuators mainly include two kinds of materials: a natural moisture sensitive material, such as agar and silk fiber, and an artificially synthesized materials, such as polyelectrolytes, conductive polymers, hydrogels, and other high molecular polymer materials. Usually, a smart actuator with humidity stimulus response can be prepared by incorporating these materials into a polymer structure. Silk fibers have good mechanical strength, dyeability, which can produce shrinkage rates that are difficult to achieve with other materials (graphene and carbon nanotube fibers). The most important is that they can provide a comfortable wearing experience and respond to humidity for the purpose of managing body temperature. Lin (Lin et al., 2020), Jia (Jia T. et al., 2019), and other researchers (Wang W. et al., 2019; An et al., 2020) adopted a conventional spinning and twisting yarn technology to prepare silk fiber actuators. Studies have shown that these types of actuators can quickly expand and contract by water absorption-induced loss of hydrogen bonds within the silk proteins and the associated structural transformation, as shown in Figure 4A. Among them, the torsional silk muscles provided a fully reversible torsional stroke of 547 mm^−1^ and 70% contraction (Jia T. et al., 2019), which is comparable to twisted CNTs fiber actuators that is widely used (Foroughi et al., 2011). And the smart textile woven from silk fiber showed sleeves of smart clothing contracted when exposed to moisture, and recovered to its original length when exposed to dry air. In addition, widely studied GR-based and GO-based actuators usually can only withstand slower bending or rotation motions under moisture stimulation. Therefore, a twisted alginate fiber-based actuator was prepared, this fiber surface transformed from smooth to a stable rough wrinkled structure when water molecules were discharged through twisting, thereby rendering a rapid and reversible rotational expansion and contraction movement (Figure 4B), which achieved a rotation speed of up to 1361 rad/s and a rotation speed of 400 turns (Wang et al., 2018c). As a new class of green materials, silk fibers and sodium alginate fiber are expected to gradually replace existing GO/GR/CNTs fiber-based actuators due to their merits in terms of low cost and good mechanical strength. So, the humidity responsive torsional artificial muscles utilizing natural textile fibers provide new ideas for natural fibers in the area of smart textile fields. Meanwhile, moisture-sensitive smart actuators based on conductive polymers have also been reported. Wang et al. combined the conductive polymer poly(3,4-ethylenedioxythiophene):polystyrene sulfonate and piezoelectric polymer PVDF by spin coating and thermal evaporation to prepare moisture-sensitive bilayer actuators (Wang G. et al., 2018), which showed a bending angle of over 180° under moisture stimulation. Moreover, owing to their mechanical displacement at different humidity levels, a generator can be prepared by connecting a piezoelectric device and these actuators, thereby producing a voltage output of 150 mV, and charging a capacitor without an energy-draining rectifier circuit, which also provided a new strategy for low-frequency small-signal energy collection and utilization. Recently, as a new 2D material, MXene (Ti_3_C_2_Tx) shows great potential as a smart humidity-responsive actuator due to its high hydrophilicity and conductivity (Wang J. et al., 2020; Nguyen et al., 2020).

### Smart Actuators Based on Thermal Stimulation Response

**FIGURE 5:**
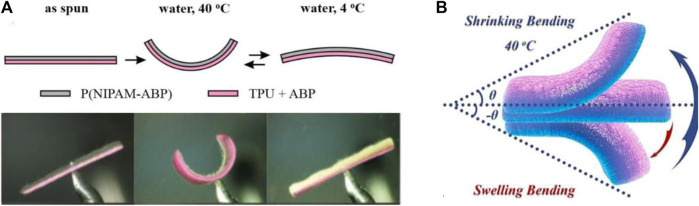
**(A)** Schematic illustration of shape of bilayer at different temperature conditions. Reproduced from Jiang et al. (2015) with permission of WILEY-VCH. **(B)** Reversible bending behavior of thermo-responsive hydrogel actuators. Reproduced from Mo et al. (2020) with permission of Royal Society of Chemistry.

Thermal responsive actuators can be divided into IR thermal drive, Joule heating drive and thermal radiation drive according to different heat sources. Among them, Joule heat driving is mainly to generate Joule heat inside the conductive material under the action of an external electric field, and then promote the material to drive deformation behavior. Thermal radiation driving is generally the driving deformation behavior that occurs under the action of an external heating source. Compared with other stimuli, thermal stimulation is safer and can achieve the corresponding actuation near living cells with the temperature between 4 and 37°C (Stroganov et al., 2014). However, the inherent disadvantage of thermal stimulus responsive smart actuators is their lower efficiency than those of different actuators based on other stimuli. In this regard, Jiang et al. photocrosslinked the thermally responsive polymer P(NIPAM-ABP) with TPU to produce a thermal stimulus-driven double layer nanofiber actuator that can quickly, reversibly, and effectively bend within 1 s at 4 and 40°C, as shown in Figure 5A (Jiang et al., 2015). In addition, using other non-reactive polymers (such as nylon 6, polysulfonamide) to replace the TPU layer can also achieve thermal stimulus response driving. In contrast to studies that improve heat conversion efficiency using more expensive raw materials, Gao et al. prepared a fiber actuator based on thermal stimulus response using low-cost hollow polyethylene with dual functional response of color and shape change (Gao et al., 2019). A fast shrinkage drive was achieved by improving the heat transfer between the materials through direct Joule heating with a shrinkage of up to 18% of the original length, thereby providing better advantages in actual industrial production. In order to further improve the response sensitivity of the actuators, Mo et al. used DC electric field to induce the gradient distribution of renewable cellulose nanocrystals (TCNs) in the PNIPAM matrix to fabricate a fast thermally responsive hydrogel for high-performance actuators (Mo et al., 2020). which achieved fast bending (4.8°/s) and recovery (1.4°/s) at 40°C and 25°C, respectively, with good fatigue resistance (Figure 5B). For thermally responsive smart actuators, increasing their responsiveness will certainly lead to more applications in soft robotics.

### Smart Actuators Based on Magnetic Stimulation Response

**FIGURE 6:**
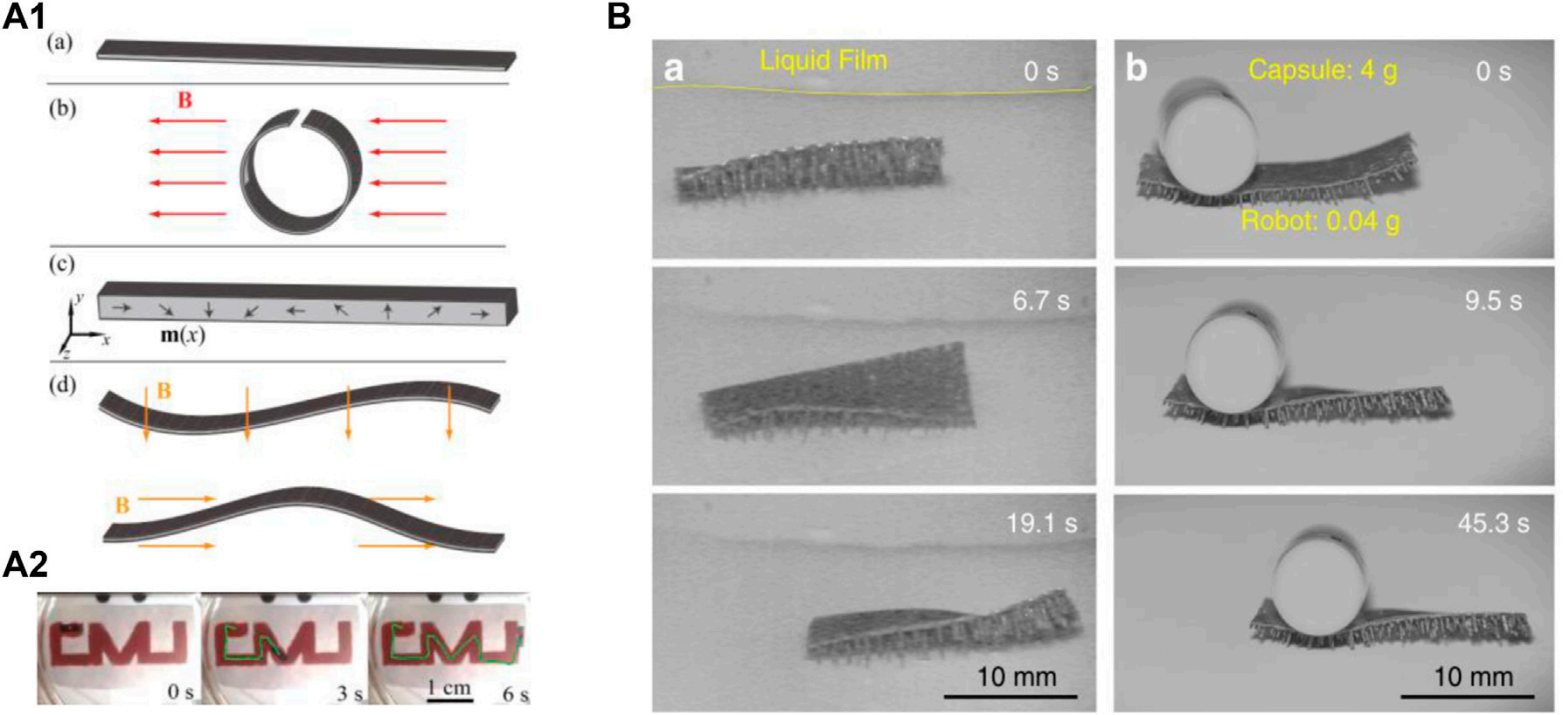
**(A1)** Continuous-magnetization-profile fabrication method, a direction varying magnetization profile is created by folding the soft materials when it is magnetized. **(A2)** Controlled path following of the robot on the water surface. Reproduced from Diller et al. (2014) with permission of AIP. **(B)** The robots move with an average speed of 0.5 mm/s on wet surface under a drive frequency of 1 Hz and move 8 mm in 45 s with a loading 100 times of its own weight. Reproduced from Lu H. et al. (2018) with permission of Springer Nature.

Soft and flexible material with magnetic particles can produce a series of shape-controllable bending and deformation behaviors in an external magnetic field. Due to the magnetic particles can make the polymer form an effective magnetic domain with variable size and direction. So, when actuators are subjected to an external magnetic field, the effective magnetic domain will be aligned along the direction of the magnetic field (Heuchel et al., 2015). And the actuator is macroscopically manifested as twisting, stretching, deformation, expansion and bending and other motion behaviors. In addition, since a magnetic field can pass through most materials magnetic stimulus responsive actuators are responsive and easy to manipulate or self-assemble, which considered to be the ideal alternative material for certain specific spatial domains theoretically (Zhao et al., 2012). At present, the research scope and application fields of magnetic responsive actuators are not as extensive as the flexible actuators described above, and they are mostly only combined with flexible polymer material or oriented magnetized to achieve magnetic response drive. Both Diller (Diller et al., 2014) and Hu (Hu et al., 2018) et al. investigated the introduction of NdFeB into different flexible polymers, and preparation magnetic responsive flexible actuators with extremely fast responsive speed (<1 s). Under an external magnetic field, the orientation of the embedded magnetic NdFeB particles completely aligned with the magnetic field direction and realize directional movement in two or three dimensions direction, as shown in Figure 6A. Lu et al. reported an unbound soft actuator (Lu H. et al., 2018), which used a modified magnetic particle-assisted molding method to enable other soft foot architectures with multiple tapered legs controlled by an external magnetic field to exhibit superior adaptability to harsh environments at ultra-fast movements (>40 limb length/s), while achieving maximum transfer capability (>100 deadweight) and excellent barrier crossing capability (90° upright, >10 body height over obstacles) (Figure 6B). Similarly, Wang et al. proposed an ultrafast response (＜0.1 s) and precisely controllable soft electromagnet actuator based on Ecoflex rubber film filled with neodymium-iron-boron (Wang X. et al., 2020). Besides, Garstecki et al. reported millimeter-scale robots can also achieve an asymmetrical swimming gait with a maximum speed of 0.3 mm/s through a rotating external magnetic field (Garstecki et al., 2009).

### Smart Actuators Based on Chemical Stimulation Response

**FIGURE 7:**
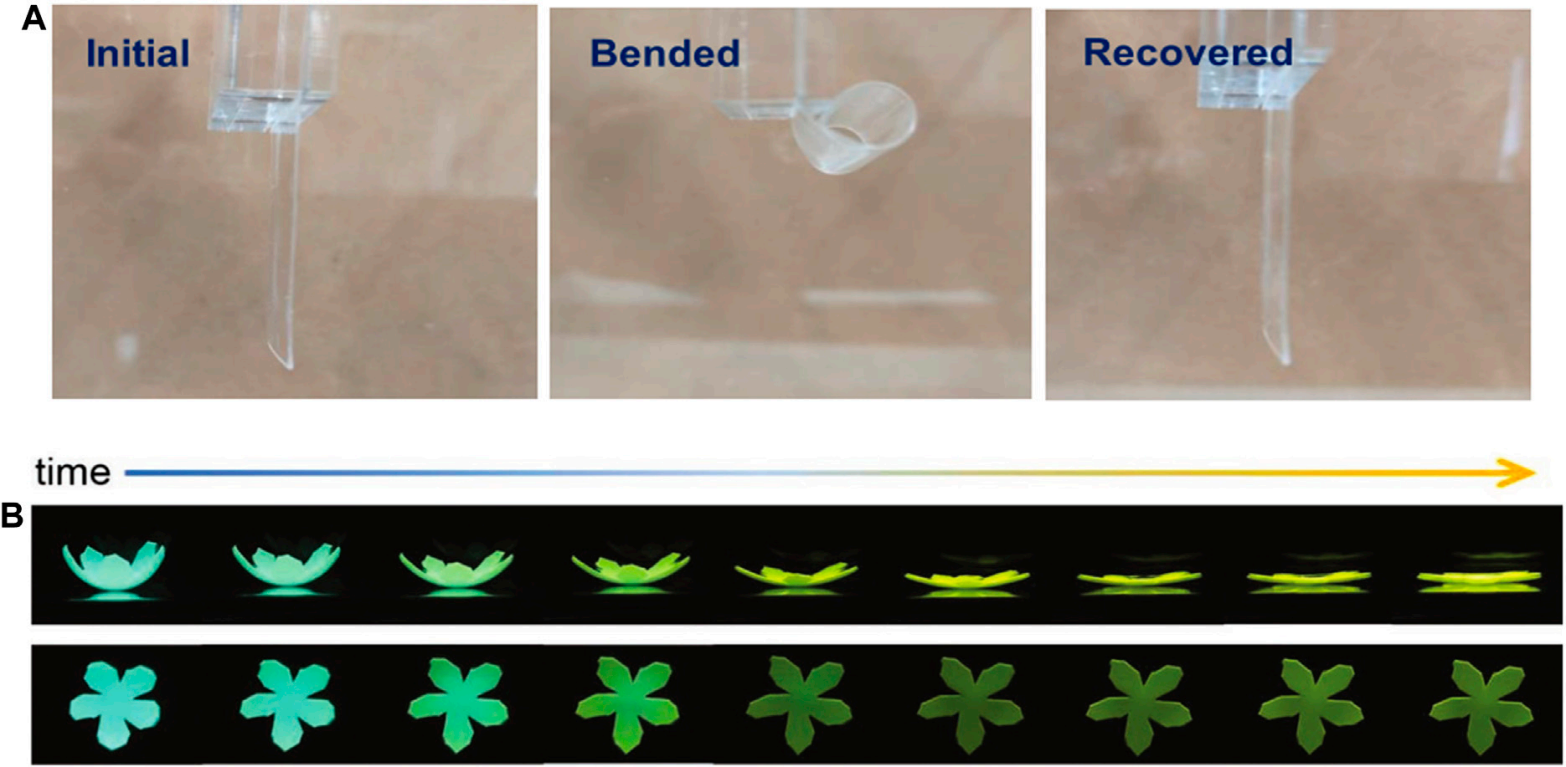
**(A)** Curling and recovery process of the PDMS film. Reproduced from Zheng et al. (2019) with permission of American Chemical Society. **(B)** Simultaneous emission change and complex shape deformation of hydrogel actuator. Reproduced from Hubbard et al. (2019) with permission of WILEY-VCH.

Chemical stimuli have a relatively wide range of influencing factors, while the response mechanism mainly includes chemical reaction induced deformation, formation and destruction of chemical bonds, and liquid action induced capillary force to produce structural deformation (Grinthal and Aizenberg, 2013). Chemical stimulus responsive driving behavior is mainly through the selective adsorption of chemical solutions by the actuator, or chemical reactions under the action of acids, alkalis, organic solvents, and water vapor to convert chemical energy into mechanical energy (Lindsey et al., 2017). Such as Hore et al. reported an elastomeric actuator, which swelled when organic solvent was added to the surface of the actuator,and thus pushing the actuator upwards with enough force to carry 10 times its own weight (Hore et al., 2012). In general, chemical stimulus actuator tend to be lower sensitive, their response time are on the order of minutes or hours. Furthermore, a small amount of chemical solvent stimulus could not easily trigger a large-scale drive behavior. In that regard, there are many studies are working to reduce the response time from minutes to seconds, UV/O_3_–modified PDMS film exhibited a series of fine wrinkles after alcohol vapor absorption for 17 s (Figure 7A), which can not only adjust the transparency of the film, but generate internal stress that trigger a large spontaneous curling deformation (Zheng et al., 2019). Gestos et al. shown microscale hydrogel fibers actuator achieving actuation strains of 20–100% and response times down to 5–10 s with pH between 3 and 8 (Gestos et al., 2012). In addition to monotonous drive changes, the chemical stimulus responsive actuator demonstrated versatile changes. Wang et al. developed an actuator that can bright color shifts and a displacement drive of 1.8 mm/s under the stimulation of chloroform, acetone, ethanol, and other organic substances (Wang et al., 2019b). Li et al. demonstrated TPE-4Py/PAS-based monolayer hydrogels and bilayer hydrogel actuators, which could simultaneously change its fluorescence color, brightness, and shape in pH 3.12, as shown in Figure 7B (Li M. et al., 2020). Unlike the traditional preparation of sandwich-structured actuators by using chemical treatments, Hubbard et al. were the first to use glass fiber fabric as the intermediate bonding phase between PDMS elastomers and polyampholytic electrolyte hydrogels, resulting in enhanced mechanical properties and better bonding of these two chemically different materials, with a bonding energy of up to 1000 N/m (Hubbard et al., 2019). This actuator achieved reversible bending behavior in salt solutions and organic solvents (e.g., acetone solutions) with drive stresses of up to 40% of the human skeletal muscle and provided new insights on the interfacial crosslinking instability common to multi-structural actuators. In addition, the combination of chemical stimulation and 3D/4D printing technology allows the easy manufacturing of arbitrarily complex configurations, such as carton panels (Zheng et al., 2018) and the “Sydney Opera House” (Huang et al., 2017). So, the anisotropic hydrogel actuator based on chemical stimulation response provides good selectivity for biological actuators, flexible robots, and other intelligent bionic device applications.

The main research progress of smart actuators based on single stimulation response is summarized in Table 1.

**TABLE 1:** Feature of multiple response smart actuator based on single stimulus responses

Actuation Method	Material	Properties	Application	References
Light	PU and CNTs	70°C NIR, contractive actuation of 6.7% (6 s), recovery (10 s).	Artificial muscle	Meng et al. (2019)
	PNIPAM and GO	0.58 mW/cm^2^ NIR, responsive swelling ratio of 6900% (4 min).	Remote light-controlled devices	Shi et al. (2015)
	PNIPAM and GO	808 nm NIR, shrinkage of 25% (360 s).	Drug delivery vehicles	Zhang et al. (2019)
	PNIPAM, RGO and PAAM	Visible light 41.8 mW/cm^2^, bending to ring (30 s); recovery (30 s).	Light-responsive actuators	Kim et al. (2016)
	Monoacrylate and Diacrylate	455 nm NIR, bending of 20 mm (3 s),recovery (8 s).	Light-driven transportation	Pilz da Cunha et al. (2020)
	GO and PC	NIR 106 mW/cm^2^, response time (<1 s), bending of 12 mm (3 s), bending curvature of 0.33 cm^-1^, recovery (5 s).	IR and sunlight-driven smart curtain, self-folding box.	Leeladhar et al. (2018)
	Sodium acrylate and IONP	2.34 W, take-off speed of 1.6 m/s (800 ms); 0.67 W, rolling velocity of 10 cm/s (1.3 s)	cargo delivery robotics	Li et al. (2020b)
	PET and Xylene	UV 170 mW/cm^2^, bending of 19 cm (14 s), recovery (4 s).	soft robotic	Verpaalen et al. (2020)
	PU, MDA and DAB	385 nm UV, 100 mW/cm^2^, bending angle of 70°(50 s).	Self-healable PME actuators	Li et al. (2019b)
Electrical	GR and PVDF	13 V, bending of 14 mm(0.26 s);17 V, Driving stress of 312.7 MPa/g, movement speed of 5.02 mm/s.	High-performance power generator	Zhao et al. (2016)
	AAm, NaAc and DMAEMA-Q	5 V/cm electric field, Bending curvature of 0.28 mm^−1^(150 s), movement speed of 2.5 mm/min.	Micro-robotics	Xiao et al. (2016)
	Graphadiyne and PVDF	2.5 V, electromechanical transduction efficiency of 6.03%, bending displacement of 16 mm.	Electro-actuation gel walker	Morales et al. (2014)
	GR and CNTs	20 mV/s voltage, tensile actuation of 19%.	Micromechanical robotics	Hyeon et al. (2019)
	GR and PANI	2.5 V, Bending curvature of 1.03 cm^-1^(5 s), areal specific capacitance of 402.5 mF/cm^2^.	Multi-functional actuator	Weng et al. (2020)
	CNTs and TEA·BF4/PC	20 mV/s voltage, tensile actuation of 16.5%, electromechanical transduction efficiency of 5.4%.	Artificial muscles	Lee et al. (2017)
Humidity	A. pernyi silk	RH 43%, rotation speed of 6179.3°/s (4.8 s), actuation power of 2.1 W/kg, contractive actuation of 10%.	Water-induced micro-actuators	Lin et al. (2020)
	Bombyx raw silk fiber	RH20% to 80%, 70% contraction, reversible torsional stroke of 547 mm^−1^	Smart textiles and soft robotics.	Jia et al. (2019b)
	Sodium alginate	RH 90%, rotation speed of 13 000 rpm (5.44 s).	Hydro-generator and breathable fabric	Wang et al. (2018c)
	PEDOT: PSS and PVDE	RH 23％ to 86％, Bending angle of 191° to 225°.	Generator and bionic field	Wang et al. (2018a)
	CS and GO	RH 45%, Bending angle of 180° (4 s).	sensors	Zhang et al. (2017)
	MXene nanosheet	RH 65%, Bending angle of 155°	flexible excavators and electrical switches	Wang et al. (2020a)
Heat	P(NIPAM-ABP), ABP and TPU	40°C, bend to ring (1 s), 4°C, recovery.	Porous 3D bioscaffolds and electrodes	Jiang et al. (2015)
	H-PE	60°C, discoloration, multiple curls (3 s), 18% contraction.	Artificial muscles	Gao et al. (2019)
	PNIPAM and TCNC	40°C, bending speed of 4.8°/s, 25 °C, recovery speed of 1.4°/s.	Temperature-controlled manipulators	Mo et al. (2020)
	CNTs, xLCE and PIM	120°C, bending curvature of 1 mm^-1^(20 s), recovery (5 s).	Restoration of deformed dynamic 3D actuators	Yang et al. (2016)
Magnetic	NdFeB and platinum-cure silicon rubber	1Hz, 2 mT, bending drive response (0.75 s), 30 Hz, 5 mT, 60 mm/s, 2.5 mT, 50 Hz, 100 mm/s.	Micro-robotics in biotechnology	Diller et al. (2014)
	NdFeB and silicone elastomer	17 mT, bending drive response (40 ms).	Soft millimetre-scale robots	Hu et al. (2018)
	PDMS and iron microparticles	200 mT, displacement of 1.2 mm, deflection angle of 18 (0.5 s).	Bio-inspired robotics	Lu et al. (2018b)
Chemical	TMPTA and DEPA	Ammonia-acetone vapor, displacement drive of 1.8 mm/s, dynamic color change of 0.16 cm/s.	Self-powered actuators and grippers	Zheng et al. (2019)
	TPE-4Py and PAS	PH3.12, Semi-circular arc expands to parallel shape and color change (400 min).	Soft robotics with communication, sensing, and disguise	Wang et al. (2019b)
	PDMS, PA and GF	Acetone, deflection angle of 48°(10 min), 2.0 M NaCl, convex (24 h).	Artificial muscles and triple-state actuators.	Hubbard et al. (2019)
	PFSA and PET	18％ Ethanol vapor, bending curvature of 0.31 mm^-1^ Deflection angle of 180° (0.25 s).	Soft actuator with multicolor switching capability	Mu et al. (2018)
	PCMVImTf 2N and PAA	1.5 mol% acetone, bending curvature of 0.38 mm^-1^.	Smart and sensitive signaling micro-robotics	Zhao et al. (2015)
	Lignin and PEGDGE	0.1 M HCl and KOH, response speed (8.0°/s) and recovery (6.5°/s),	Flow control valve and smart hook	Dai et al. (2020)

## Multiple Stimuli Response Smart Actuators

Although single stimulus response smart actuator can be precisely controlled and has a relatively simple preparation, the actual environment is highly diverse and complex with more than a single stimulus source. Therefore, single stimulus response or simple functional output of the above smart actuators are no longer sufficient for the current actuation requirements in complex environments. The development of newer stimulus conditions and diversified stimulus methods has become an urgent requirement for stimulus-responsive smart actuators (Wang T. et al., 2018). Generally, multi-stimulus response smart actuators are mainly constructed by introducing multifunctional stimulus responsive groups into polymeric materials, achieving multi-responsive properties through precise molecular design or blending methods (Deng et al., 2015) (Cheng et al., 2016).

### Smart Actuator Based On Dual Light And Heat Stimuli Response

**FIGURE 8:**
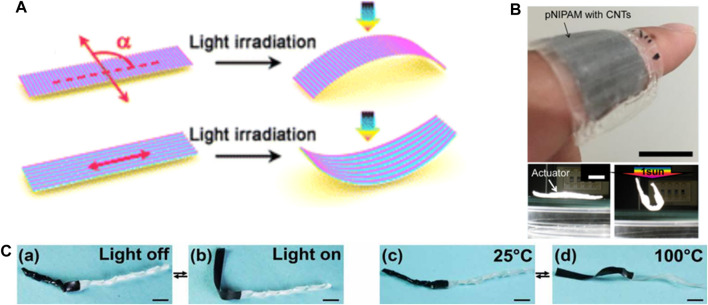
**(A)** Schematic illustration of the apheliotropic and phototropic bending of the composite strips with different aligned directions of smart actuator. Reproduced from Deng et al. (2015) with permission of American Chemical Society. **(B)** Demonstrations of the PNIPAM actuators stimulated by the human skin temperature and sunlight. Reproduced from Yamamoto et al. (2015) with permission of American Chemical Society. **(C)** Schematic showing of an area-selective reversible multiresponsive actuator. Reproduced from Zhang Y. et al. (2020) with permission of American Association for the Advancement of Science.

Currently, some photo-thermal conversion effect materials (GR, GO, CNTs, PDA) are added to some thermally responsive shape memory polymer materials, which can produce thermal effects under the irradiation of light to achieve light/thermal dual stimulation. Among them, PA6 has high spinnability and hygroscopicity, the fiber actuators with spiral structures and light/heat response can be obtained by electrospinning and twisting treatment. Huang et al. (Huang et al., 2020) added PDA before twisting, resulting in a driving stress of approximately 0.9MPa and shrinkage rate of 5.1% under NIR light and 180°C. Yamamoto et al. combined CNTs with PNIPAM as photothermal conversion materials of their prepared actuators that could achieve a bending deflection of 210° within 80 s under photothermal stimulation (Figure 8B) (Yamamoto et al., 2015). However, its response time was lower than that of the PNIPAM/GO photothermal response actuator prepared by in situ polymerization and centrifugal method (He et al., 2019), which could also achieve rapid and controllable bidirectional bending within 30 s. In addition, the double-layer thin-film smart actuator composed of paraffin wax and CNTs can also achieve the corresponding dual stimulus response bending behavior (Deng et al., 2016) (Figure 8A). MoS_2_ nanosheets can enable the actuator to achieve adjustable light and heat response drives when incorporated in hydrogel carboxyl chitosan as light and heat transfer agent (Lei et al., 2016). The anisotropic structure of the actuator allowed good shape deformation and self-wrapping kinematic properties by the remote control of NIR light or temperature of 70°C and recovery to its initial state in a relatively short time at room temperature. Meanwhile, Zhang et al. proposed that different types of liquid crystal elastomer materials can be cross-linked with functional media while being oriented to prepare composite flexible actuators with different driving modes under light and thermal stimulation, showing the highest driving strain (Figure 8C) (Zhang Y. et al., 2020). This kind of light/heat dual stimulus responsive actuators will have broad development prospects in smart machinery and other fields.

### Smart Actuator Based on Dual Light and Electric Stimuli Response

Smart actuators based on light and electric stimuli response can be prepared by combining light-sensitive materials with electroactive polymers. However, a common problem is lower curvature for such actuators (Seo et al., 2012). Therefore, Yang et al. (Wei et al., 2020) reported a sericin functionalized RGO (SRGO)/ PI double layer actuator with light and thermal stimuli response by directly coating RGO paper on PI tape. Owing to the deformation of the micro-airbags in the SRGO layer and thermal expansion of the PI layer, the actuator can achieve bending deformations of 0.55 cm^-1^ under 16 V or light stimulation. The photo-mechanical drive and triboelectric effect of the integrated SRGO/PI double layer actuator was used to assemble a photoelectric generator. Similarly, Weng et al. also prepared a light-electric dual stimulus-response actuator based on high-efficiency conduction of GR and thermal conversion effects, which can produce up to 2.6 cm^-1^ bending drive behavior for NIR light and electrical stimulation (Weng et al., 2016).

### Smart Actuator Based on Dual Light and Magnetic Stimuli Response

**FIGURE 9:**
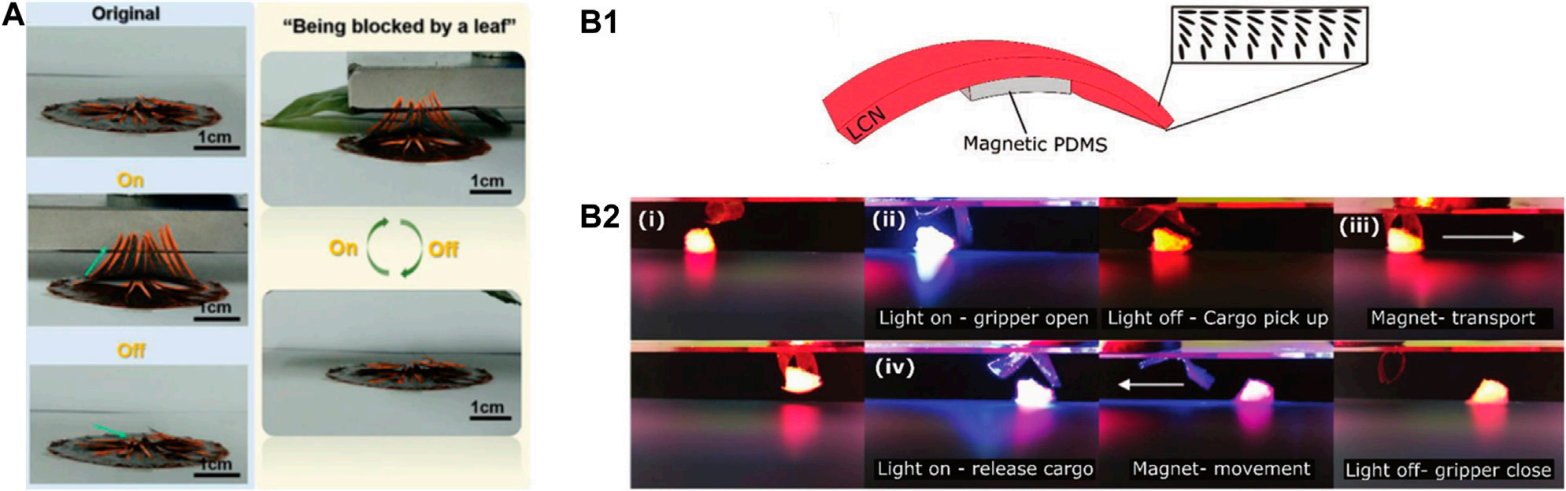
**(A)** Photographs of a “mimosa pudica” splaying and closing, and morphing blocked by a leaf and exposed to magnetic field, respectively. Reproduced from Wang et al. (2019c) with permission of WILEY-VCH. **(B1)** Magneto-light responsive actuator with localized PDMS/Fe composite layer coated on LCN. **(B2)** The untethered pick up, transport, and release of a cargo, performed by the dual-responsive gripper within an enclosed space. Reproduced from Pilz da Cunha et al. (2019) with permission of WILEY-VCH.

Generally, opto-magnetic response actuators can be simply obtained by incorporating magnetic nanoparticles to a light-responsive actuator (Cheng et al., 2017; Gelebart et al., 2017). Such as, Fe_3_O_4_NPs can make composite materials magnetic, Wang et al. introduced Fe_3_O_4_/CNC nanocrystal nanohybrids as the response medium to presents a superfast magnetic response of 0.36 s and light response of 0.44 s (Wang et al., 2019c), as shown in (Figure. 9A). The metal ligand coordination between Fe_3_O_4_ NPs and the catechol groups of DOPAC achieved an ultra-high photothermal conversion efficiency of 79.1% by crosslinking interfacial supramolecule and DOPAC acid. However, Han et al. asymmetrically distributed Fe_3_O_4_ NPs in RGO to alter their water absorption capacity, resulting in the stimulus responses to light, heat, water, and magnetic conditions (Han et al., 2020). This also solved the problem of interlayer separation in a dual piezoelectric wafer actuator. Furthermore, the flower-shaped actuator could perform a simple co-bending drive in a complex environment, where multiple stimuli simultaneously exist. Recently, Pilz et al. combined PDMS layer functionalized with carbonyl magnetic iron powder and the LCN containing a photosensitive azobenzene dye, as shown in Figure 9B, which achieved a breakthrough in the uniformity of the drive under dual stimulation in the same space (Pilz da Cunha et al., 2019). The azobenzene derivative was rapidly isomerized and generating heat to realize the bending and capturing behavior of the actuator under light stimulation, while the magnetic response was used as a magnetic guide to drive the actuator with translational and rotational degrees of freedom (Figure 9B).

### Smart Actuator Based on Dual Heat and Chemical Stimuli Response

Since the ionizable acid groups in PAA hydrogel can accept and deliver protons in response to changes in pH. Thus, Shang et al. added PNIPAM with a high expansion and temperature sensitivity to PAA hydrogel (Shang and Theato, 2018a), the bilayer hydrogels show a reversible and repeatable direction-controllable curving behavior upon variation of temperature (2-50°C) and pH (2 and 11). At the same time, by combining the inhomogeneity of lateral hydrogel composition (PAA and PNIPAM/PAA) and dimensions (size of PAA and PNIPAM/PAA region), a complex 3D deformation also could be generated. By replacing PAA with 2-carboxyethyl acrylate, the actuator can also respond to ethanol vapor at a slower response speed (Odent et al., 2019). Subsequently, in order to further narrow the temperature difference range of the actuator response, Li et al. prepared an semi-interpenetrating network hydrogel-based bilayer actuators by generating a PNIPAM-based hydrogel in the presence of positively charged polyelectrolyte pDADMAC on a layer of gold-coated PDMS, which showed quickly bi-directional bending behavior in response to solution temperature(25-45°C) and PH(3 and 6.5) (Li et al., 2017b). To address the weak mechanical properties of hydrogel actuators, heat-chemical response hydrogel actuators composed of SMA, AA, and QCH utilize the electrostatic interaction between AA and QCH, and hydrophobic interaction of alkyl chains in SMA to provide a high strain stress (906%, 1.64 MPa) and fatigue resistance (Jing et al., 2019). Soon afterwards, based on electrostatic spinning technique, a smart actuator with high mechanical properties was obtained by combining submicron particles of PNIPAM and chitosan into a structure of PLA microfibers, which also showed temperature and pH responsiveness (Štular et al., 2019). Therefore, these actuators have flexible design and is widely used in the field of flexible actuators, even in the field of bionic robots.

### Smart Actuator Based on Multiple Light, Heat, and Humidity Stimuli Response

GO with oxygen-rich groups is the ideal material for multi-stimuli response actuators. GO–CNT/PDMS double layer film actuator was prepared by embedding a PDMS layer with CNT strips (Wang et al., 2018d). Under light stimulation, the response time of this actuator was longer than that of a GO film actuator prepared by GO suspension casting (Cheng et al., 2016), which has a better humidity bending drive response of 137°. Recently, Zhang et al. further adopted nanoscale graphite, by combining a composite layer of Nano-G and PVDF with GO to achieve bidirectional drive under humidity and light stimulations, further manufacturing a bidirectional walking robot, which the average moving speeds are 0.4 mm/s and 1 mm/s for moisture and light actuation, respectively. (Zhang Y.-L. et al., 2020). Chen et al. also proposed an actuator based on GO and biaxially oriented polypropylene composites that utilized the wet expansion and photothermal conversion properties of GO, allowing the actuator to achieve a bending curvature of up to 3.1 cm^−1^ under humidity stimulation, which is higher than that under light stimulation (Chen et al., 2017). In addition, actuators prepared by coating a highly hygroscopic film (pyrolytic graphite) on an antimagnetic graphite film, which can also realize high-speed linear motion (88 mm/s) and turning motion (180°/s) under the IR light and humidity (Ji et al., 2020). Above all, the excellent electrical, mechanical, and thermal properties of graphene enable it to be widely used in materials and structural components of multiple smart actuators.

The main research progress of smart actuators based on multiple stimuli responses are summarized in Table 2.

**TABLE 2:** Feature of multiple response smart actuator based on multiple stimulus responses

Actuation Method	Material	Properties	Application	References
Light/Heat	PA6 and pDA	180°C, contractive actuation of 5.1%. NIR, contractive actuation of 3.2%.	Artificial muscle	Huang et al. (2020)
	MoS2 and Carboxyl	70°C, 5 W/cm^2^ NIR, bending curvature of 0.23 cm^-1^ (70 s).	Flexible anisotropic actuator	Lei et al. (2016)
	PNIPAM and CNTs	50°C, bending angle of 210° (80 s). 100 mW/cm^2^ light, bending angle of 210° (14 min).	Wearable device and natural power source actuator	Yamamoto et al. (2015)
	PNIPAAm, BIS and PBPO	35°C, bending curvature of 5.2 cm^-1^ (7 min). 665 nmUV, red fluorescence appears.	Biomimetic devices, gripper, and information storage	Zhang et al. (2020b)
	PNIPAM and GO	55°C, bending angle of 210° (16 s). 2.5 W/cm^2^ NIR, curl to closed state (17 s).	Remotely controlled microgrippers	He et al. (2019)
	GO and MAB	100°C, reversible spiral bending. NIR, Curly flattening.	3D machine- and animal-mimicking LCE actuators	Zhang et al. (2020d)
	EDDET, PMMS and PETMP	90°C, bending angle of 80°. 0.8 W/cm^2^ NIR, bending angle of 110° (15 s). 80 W/cm^2^UV, bending angle of 100° (11 s).	Artificial plants, and multiple-responsive microrobots	Zhang et al. (2020a)
Light/ Electricity	RGO and PI	16 V bending curvature of 0.55 cm^-1^ (5 s). 300 mW/cm^2^ light, bending curvature of 0.45 cm^-1^ (10 s).	Electrothermal actuator, microfluidics	Seo et al. (2012)
	GR and BOPP	10 V bending curvature of 2.6 cm^-1^. 300 mW/cm^2^ NIR, bending curvature of 1.9 cm^-1^ (10 s).	Biomimetic flower, and smart household materials.	Weng et al. (2016)
	SWCNT and PE	9.0 V, bending curvature of 7.8 cm^-1^ (3 s). NIR 250 mW/cm^2^, bending curvature of 5.0 cm^-1^ (3 s).	Walking device, smart mechanical devices	Li et al. (2018)
Light/magnetic	PU, DOPAC and Fe3O4NPS	Maximum bending angle, 808 nm NIR (0.44 s). magnetic field (0.36 s).	Bionic motion robots	Wang et al. (2019c)
	Fe3O4NPs and GO	200 mW/cm^2^ light, bending angle of 210° (30 s). NdFeB Magnetic field, bending angle of 90°(3 s).	Multi-form actuators with different fields	Han et al. (2020)
	PDMS, DCM and Acrylate	225 mW/cm^-2^ light. Deflection displacement of 14 mm (10 s). Magnetic field, grasping or bending behavior.	Dual-responsive gripper, soft robotics with programmed	Pilz da Cunha et al. (2019)
Heat/chemical	PNIPAM and PAA	2°C and 50°C, PH 2 and PH 11, two-way bending, bidirectional bending drive	Temperature-induced self-bending actuators	Shang and Theato (2018b)
	PNIPAM, CEA and MBA	Ethanol solution, bending curvature of 1.4 cm^-1^ (90 s). PH3, bending curvature of 1.75 cm^-1^ (45 min), PH8, recovery (20 min). 50°C, bending curvature of 1.55 cm^-1^.	Anisotropy-encoded hydrogel actuators, dual-responsive grippers	Odent et al. (2019)
	PDADMAC, PNIPAM and PDMS	25°C and 40°C, PH 6.5 and PH 3, downward spherical bending to upward bending, shrinkage rate 60%(10 min).	Stimulus-induced grippers, biomedical applications field	Li et al. (2017a)
	P(NIPAM-coAAC) and NaAlg	50°C, 1M CaCl2, Shrinkage deformation rate 20%(200 s).	Chemical sensors, microengineering	Yoshida et al. (2018)
	SMA, AA and QCH	80°C, spiral state(3 s).	Soft robotics with programmable combination	Jing et al. (2019)
		Curly recovery under alkaline and acidic conditions		
	PNIPAAm, PNCS and PLA	20°C and 40°C, PH 3 and PH 8, shrink/expansion response	Artificial muscle	Štular et al. (2019)
	CNT and PDMS	225°C, bending curvature of 0.3 cm^−1^. Potassium chloride solution, displacement of 4 mm(4 s)	Crawling robot like an inchworm, a gripper to grasp	Ji et al. (2019)
	Dns and PAAM	PH 11.5 and PH 2.0, bend and return to original state; 50°C, recovery.	Soft robots	Gong et al. (2016)
Light,heat,and humidity	GO, CNTs and PDMS	0.5 w/cm^2^ light, bending angle of 90° (2.5 s).	Biomimetic devices, humidity control switches, and optical control medical devices	Wang et al. (2018d)
		80°C, bending angle of 180° (1.7 s).		
		RH 90％, bending angle of 137° (1.4 s).		
	GO	IR light and 100°C, bending angle of 90° (1 s).	Multifunctional smart walkers with self-deformation sensing ability	Cheng et al. (2016)
		RH 85%, bending angle of 70° (1 s).		
	Nano-size graphite, PVDF and GO	206 mW/cm^2^ light and 70°C, bending angle of 160° (4 s). RH 23% to 97%, bending angle of 200° (13 s).	Multi-responsive Bimorph actuators, smart claw	Zhang et al. (2020c)
	GO and BOPP	80°C, 300 mW/cm^2^ light, bending curvature of 2.8 cm^-1^. RH 20% to 90%, bending curvature of 3.1 cm^-1^.	Artificial muscles, bioinspired robotics	Chen et al. (2017)
	PG and graphite	RH 70%, curvature change speed of 1 cm^−1^s^−1^.	Soft robotics and smart mechanical devices	Ji et al. (2020)
		1.2 W/cm^-2^, curvature change speed of 1 cm^−1^s^−1^.		

## Conclusion

In summary, the design concept of stimulus-response smart actuators mainly comes from organisms in nature, which is to prepare actuators with similar stimulus responses by observing behavioral characteristics of life forms. Most of current ongoing research involves the biocompatible actuators that can operate under multiple stimuli conditions. Among them, GO, CNTs, and other materials with high thermal expansion properties (such as Dns and p-phenylenediamine) are used as functional dielectric materials. Elastomeric polymer materials with good biocompatibility, such as common PDMS, PVDF, and PNIPAM, are commonly used as the basic flexible materials that can respond to light, heat, electricity and chemical stimuli. However, most of the these actuators are double-layered structures and face the problems of instability and easy separation between boundary layers. In recent years, fiber-based actuators with a spiral structure have received widespread attention because of their mechanical strength and multi-response editability. But they have only been applied in the fields of light, heat, and humidity stimulus response. Correspondingly, chemical stimulus response smart actuators have unique functional changes that are not yet applicable to other stimulus response actuators, their slow response time limits their application. Despite these limitations, it is expected that the stability and responsiveness of smart actuators will continue to increase, enabled by the discovery of responsive materials with high photothermal conversion efficiency and multi-functionality. Moreover, porous fiber microstructures and various spun fiber structures can be used to enhance the transport of swollen or contracted water molecules and increase the response speed. Thus, it is expected that there will be broad application prospects and high added value in the fields of smart robots, artificial muscles, biological sensing, and smart medical equipment in the future.

## Abbreviations:

AA: Acrylic acid; CNT: Carbon nanotube; DOPAC: 3,4-dihydroxyphenylacetic acid; GO: Graphene oxide; LDAM: Light-driven artificial muscle; Nano-G: Nanoscale graphite; NIR: Near-infrared; NP: Nanoparticle; PAA: Polyacrylic acid; PDA: Polydopamine; PDMS: Poly(dimethyl siloxane); PI: Polyimide; PNIPAm: Poly(N-isopropylacrylamide); P(NIPAm-ABP): Polymer poly(N-isopropylacrylamide)-acryloylbenzophenone copolymer; PU: Polyurethane; PVDF: Polyvinylidene difluoride; QCH: Quaternary ammonium chitosan; RGO: Reduced graphene oxide; SMA: Octadecyl ester; SRGO: Sericin-functionalized reduced graphene oxide; TPE-4Py: Tetrakis (4-pyridinephenyl) ethylene; TPU: Thermoplastic polyurethane; GP: graphite/polyaniline; PEGDGE: poly(ethylene glycol) diglycidyl ether.
